# StrAuto: automation and parallelization of STRUCTURE analysis

**DOI:** 10.1186/s12859-017-1593-0

**Published:** 2017-03-24

**Authors:** Vikram E. Chhatre, Kevin J. Emerson

**Affiliations:** 1 Department of Plant Biology, University of Vermont, Burlington, Vermont USA; 2 Current Address: Wyoming INBRE Bioinformatics Core, Department of Molecular Biology, University of Wyoming, Laramie, Wyoming USA; 3 Department of Biology, St. Mary’s College of Maryland, St. Mary’s City, Maryland USA

**Keywords:** STRUCTURE analysis, Parallelization, Population genomics

## Abstract

**Background:**

Population structure inference using the software STRUCTURE has become an integral part of population genetic studies covering a broad spectrum of taxa including humans. The ever-expanding size of genetic data sets poses computational challenges for this analysis. Although at least one tool currently implements parallel computing to reduce computational overload of this analysis, it does not fully automate the use of replicate STRUCTURE analysis runs required for downstream inference of optimal *K*. There is pressing need for a tool that can deploy population structure analysis on high performance computing clusters.

**Results:**

We present an updated version of the popular Python program StrAuto, to streamline population structure analysis using parallel computing. StrAuto implements a pipeline that combines STRUCTURE analysis with the Evanno *Δ* *K* analysis and visualization of results using STRUCTURE HARVESTER. Using benchmarking tests, we demonstrate that StrAuto significantly reduces the computational time needed to perform iterative STRUCTURE analysis by distributing runs over two or more processors.

**Conclusion:**

StrAuto is the first tool to integrate STRUCTURE analysis with post-processing using a pipeline approach in addition to implementing parallel computation – a set up ideal for deployment on computing clusters. StrAuto is distributed under the GNU GPL (General Public License) and available to download from http://strauto.popgen.org.

## Background

Inference of population structure has found application in fields as varied as human genetics (e.g., [1]) evolution and speciation [2], molecular ecology [3], landscape genetics [4], agriculture [5], forest population genomics [6], tree improvement [7], fisheries [8] and many others. The Bayesian algorithm implemented in the software STRUCTURE [9–11] is now among the most heavily used methods to infer population structure from genotype data despite the difficulties in making unbiased estimates of population structuring under various models of demograhic history [12] or when not using balanced population sampling [13]. The STRUCTURE algorithm is a model-based clustering method that uses Markov Chain Monte Carlo (MCMC) re-sampling to determine the likelihood of a particular number of Hardy-Weinberg linkage equilibrium clusters (*K*) for a given genotype dataset. Once several replicate analyses for a variety of *K* values are completed, one can then determine the optimal number of inferred genetic clusters (*K*) that individuals within a given dataset draw ancestry from. This is most commonly accomplished using the Evanno method [14].

Due to the underlying nature of the MCMC process, the STRUCTURE algorithm is computationally intensive while requiring very little computer memory. A single run of the algorithm can take 100% of a processor’s computing power for several hours to complete. This, along with the replication required to generate the tests of likelihoods, can lead to a single analysis taking between several days to weeks to complete, even with fewer-than-optimal number of replicates being performed.

The ever-expanding size of population genetic data sets generated by next generation sequencing technologies and other high-throughput genotyping platforms (e.g., [15, 16]) presents an additional significant computational challenge for researchers interested in performing STRUCTURE analyses. Phylogeographic datasets, one class of dataset for which STRUCTURE analysis is common, have shown a drastic increase in size over the past five years, with some projections showing that the median number of Single Nucleotide Polymorphisms (SNPs) per dataset may approach 20,000 by the end of 2016 [16]. Recently a new method - fastSTRUCTURE [17], was developed to speed up inference of population structure in large genome-scale data sets, but it leverages approximation to make computational gains at the cost of user directed model selection. Given the continuing popularity of original STRUCTURE algorithm, it is highly likely that researchers interested in model selection will continue to use it even with genome-scaled data sets. Such analyses would significantly benefit from taking advantage of parallel computing in multi-core computing environments using a streamlined pipeline - beginning with the replicated STRUCTURE runs and ending in collation of results using the pre-existing script STRUCTURE HARVESTER [18] that is designed to visualize STRUCTURE output and to implement the Evanno method [14] to determine the optimal number of clusters (*K*).

Large datasets also make it more difficult to perform exploratory analysis of datasets that inform the full and complete analysis. As the documentation for STRUCTURE correctly points out, “...some care is needed in running the program in order to ensure sensible answers. For example, it is not possible to determine suitable run-lengths theoretically, and this requires some experimentation on the part of the user.”(STRUCTURE software manual 2.3.4). In the case of large data sets, this process is often challenging due to the time required to perform a single run of the STRUCTURE algorithm. Tools to enhance the speed of these initial exploratory runs as well as the full STRUCTURE analysis would be very useful.

While STRUCTURE can be easily implemented using the back-end command-line interface, it lacks the front-end functionality of setting multiple iterative runs to test *K* clusters and then collating the data from each run. STRUCTURE is also not designed to make use of parallel computing, now commonly available on personal computers and high performance computing (HPC) clusters. Although runs of STRUCTURE have been parallelized through the R programming language (R core team 2015) package - PARALLELSTRUCTURE [19], it requires that individual iterative runs for each *K* cluster being tested be manually specified on the *joblist*. Additionally, some proficiency in the R programming language is required to make use of this package.

To address these issues, we present an updated version of the popular stand-alone, interactive, Python program StrAuto to automate and parallelize STRUCTURE analysis on Macintosh OS and the various flavors of Unix running on workstations and HPC clusters. The use of the script requires no knowledge of Python programming, and only requires basic interaction with the UNIX command line. This program also includes a secondary script sampleStructureFile that randomly samples loci from a structure file so that researchers can use subsets of their data (e.g. 10% of loci) for initial exploratory experimentation before they commit longer periods of time to the full analysis. We demonstrate the usage and benchmark the results from analysis of two example data sets on a standalone computer and a HPC cluster. StrAuto version 1.0 is available for download from http://strauto.popgen.org.

## Implementation

The StrAuto workflow is as follows:

1. Information about the user project and intended analysis, including the fraction (or number) of available processing cores to be committed for parallelization, is collected from a template text file.
2. Using this information StrAuto outputs a Unix shell script (runstructure), two parameter files required by STRUCTURE, and if parallelization is chosen, a file with all individual STRUCTURE commands (structureCommands).
3. Optional parallelization is implemented through GNU Parallel [20] (http://www.gnu.org/software/ parallel) which should be installed locally.
4. Upon execution, the shell script ‘runstructure’ runs STRUCTURE for *K* clusters over *n* iterations distributed over *x* number of processors.
5. Results are compiled into a zip file and fed through STRUCTURE HARVESTER [18], if available locally.
6. Final output is ready for visualization and inference of population structure.

Also included with StrAuto is the script sample StructureFile, which takes a structure-formatted datafile and randomly samples a given number of loci in to a new file for use in initial exploratory experimentation. More information on the use of this script is available in the StrAuto user manual.

### Speed benchmarking trials

In order to benchmark StrAuto’s ability to speed up STRUCTURE analysis on parallel computing platforms, it was tested on two different data sets. The first data set (SSR) included genotypes at 11 nuclear microsatellite loci from 614 eastern white pine trees (*Pinus strobus*) [21], and the second (SNP) data set included genotypes from 799 nextRAD based SNP loci from 57 individuals of the Neotropical malaria vector *Anopheles darlingi* [22]. StrAuto was used to set up the STRUCTURE analysis with *K* varying from 1 to 8 (SSR) and 1 to 5 (SNP) and with MCMC chains of 1.1 million generations with the first 100,000 generations discarded as burnin. This led to a total of 80 (SSR) and 50 (SNP) independent runs of the program STRUCTURE (v. 2.3.4). To benchmark the computational gain achieved using StrAuto, we replicated all analyses on two separate systems. A standalone server with 64 cores and 196 Gb of physical memory running Ubuntu Linux version 14.04 was set up to incrementally use 1, 5, 10, 20, 30 and 50 cores. A research computing cluster (HPC) with multiple nodes, each with 16 cores and 128 Gb of memory running Red Hat Enterprise Linux version 6.7 [23] was set up to incrementally use from 1 to 6 nodes (1, 16, 32, 48, 64, 80 and 96 cores respectively). The SSR data (80 processes) was run on up to 30 cores on the standalone computer and 96 cores (6 nodes) on the HPC cluster. The SNP data (50 processes) was run on up to 50 cores on the standalone computer and 64 cores on the HPC cluster.

## Results and discussion

**Fig. 1:**
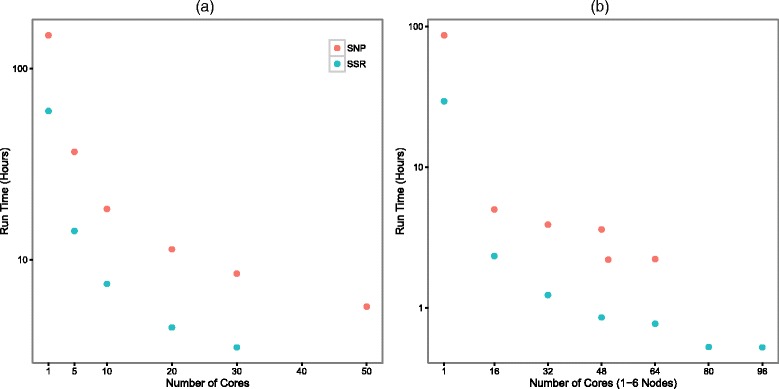
Speed benchmarking for the analysis of two trial datasets incorporating various numbers of cores on two different computing systems. Single Nucleotide Polymorphisms (SNP) data is from [22] and Simple Sequence Repeat (SSR) data is from [21]. The asymptotic approach to a minimum time represents the time it takes for a single core to compute one complete run of the program STRUCTURE for the given dataset. **a** Standalone Computer. **b** HPC Cluster

In the benchmark trials, there was a drastic decrease in the total analysis time with increasing number of cores applied (Fig. 1), regardless of whether the analysis is of a large dataset with a few loci genotyped in many individuals (SSR) or many loci genotyped in a few individuals (SNP). It is important to note that StrAuto is not parallelizing the STRUCTURE algorithm directly, but is distributing the replicate runs of the STRUCTURE algorithm to different processor units. Therefore, any computational gains are determined primarily by the number of cores available and the total length of time required for a single STRUCTURE run. One might even argue that StrAuto will potentially lead to more ‘accurate results’ in shorter amount of time merely by empowering users to (1) run sufficient numbers of replicate runs and (2) perform an appropriate number of burnin and MCMC sweeps, both necessary for proper inference of the optimal *K* solution, than if they were limited to running only one instance at a time. StrAuto allows users to efficiently leverage the computational power of their hardware for this analysis. For instance, analysis of the SNP dataset using a single core, which included 50 independent runs of the STRUCTURE algorithm, took 149.2 h to complete on the standalone computer and 86.4 h on the HPC cluster (Fig. 1). Using 50 separate cores, one per independent run of STRUCTURE, the total time to complete the analysis was 5.68 h on the standalone computer (∼26 times faster) and 2.22 h on the HPC cluster (∼39 times faster). Analysis of the SSR data set on the HPC cluster took 29.38 h to complete using one core and 32 min using 80 cores (∼56 times faster; Fig. 1 b). The scaling seen using StrAuto will be dependent upon the architecture of the computing environment. Interdependence of available cores and overhead costs involved in parallel processing will lead to less performance gain than when cores are running independently. Other factors such as disk I/O requirements of the program and hyperthreading may also affect the scaling. Therefore one should not expect linear scaling of the time needed for the analysis. However, because the runs of STRUCTURE are independent from one another, one will always see a speedup of total computation time as one increases the number of cores available for computation – until the number of cores exceeds the total number of independent STRUCTURE runs. This is clearly evident from our benchmarking tests which show no further speedup once the number of cores exceed total number of runs e.g. SNP analysis using 50 *vs* 64 cores and SSR analysis using 80 *vs* 96 cores (Fig. 1 b). On the other hand, it took 1.41 h of additional computation time to complete analysis of the SNP data set (total 50 runs) using 48 *vs* 50 cores. This is because when using 48 cores, the analysis must wait for two cores to become available before proceeding with 49th and 50th independent run (Fig. 1 b). There is no wait when using 50 cores because the number of runs is equal to number of cores engaged.

A larger dataset from [22], with 11,533 loci genotyped among 57 individuals, that analyzed 30 replicates for each *K* ranging from 1 to 6 with MCMC chains as defined above, took just over 9 days to complete on 60 cores using the StrAuto script. Initial exploratory experimentation to determine the MCMC parameters with this dataset was conducted using 500 randomly sampled loci, and took ∼4 h with 30 cores.

In summary, the time to complete a fully replicated STRUCTURE analysis is a function of the number of cores available and the time to complete a single run of the algorithm. Users with access to smaller numbers of cores may consider using other multi-core or cloud-based computing platforms when analyzing large datasets. As our results show, HPC clusters offer greater scalability for this analysis with up to 56 times speedup with our example data than standalone computers which showed upto 26 times speedup.

## Conclusions

StrAuto is the first tool to implement a pipeline approach by (1) combining STRUCTURE analysis with downstream collation of results using STRUCTURE HARVESTER, and (2) distributing runs over multiple processors using GNU Parallel. These functionalities make StrAuto ideal for deployment on high performance computing clusters and multi-core personal workstations, to reduce the computational time.

## Abbreviations

Gb: Giga Bytes
GNU: GNU’
s not Unix; GPL: General Public License
HPC: High Performance Computing
I/O: Input/Output
Macintosh OS: Macintosh Operating System
MCMC: Markov Chain Monte Carlo
nextRAD: Next Generation Restriction Site Associated DNA Sequencing
SNP: Single Nucleotide Polymorphism
SSR: Simple Sequence Repeat
